# Phylogeography of *Francisella tularensis* subspecies *holarctica* and epidemiology of tularemia in Switzerland

**DOI:** 10.3389/fmicb.2023.1151049

**Published:** 2023-04-11

**Authors:** Sara Doina Schütz, Nicole Liechti, Ekkehardt Altpeter, Anton Labutin, Tsering Wütrich, Kristina Maria Schmidt, Michael Buettcher, Michel Moser, Rémy Bruggmann, Matthias Wittwer

**Affiliations:** ^1^Interfaculty Bioinformatics Unit, University of Bern and Swiss Institute of Bioinformatics, Bern, Switzerland; ^2^Spiez Laboratory, Federal Office for Civil Protection and Swiss National Reference Center for Highly Pathogenic Bacteria (NABA), Spiez, Switzerland; ^3^Graduate School for Cellular and Biomedical Sciences, University of Bern, Bern, Switzerland; ^4^Swiss Federal Office of Public Health, Bern, Switzerland; ^5^Paediatric Infectious Diseases Unit, Children’s Hospital Lucerne, Lucerne Cantonal Hospital, Lucerne, Switzerland; ^6^Faculty of Health Sciences and Medicine, University of Lucerne, Lucerne, Switzerland; ^7^Paediatric Pharmacology and Pharmacometrics Research Center, University Children’s Hospital Basel, Basel, Switzerland

**Keywords:** *Francisella tularensis* subsp. *holarctica*, canSNPs, epidemiology of infectious diseases, tularemia, whole-genome sequencing, antimicrobial susceptibility testing

## Abstract

Tularemia, an endemic disease that mainly affects wild animals and humans, is caused by *Francisella tularensis* subsp. *holarctica* (*Fth*) in Switzerland. The Swiss *Fth* population consist of multiple different subclades which are distributed throughout the country. The aim of this study is to characterize the genetic diversity of *Fth* in Switzerland and to describe the phylogeographic relationship of isolates by single nucleotide polymorphism (SNP) analysis. This analysis is combined with human surveillance data from reported cases over the last 10 years and *in vitro* and *in silico* antibiotic resistance tests to provide insight into the epidemiology of tularemia in Switzerland. We sequenced the whole genomes of 52 *Fth* strains of human or tick origin collected in Switzerland between 2009 and 2022 and analyzed together with all publicly available sequencing data of Swiss and European *Fth*. Next, we performed a preliminary classification with the established canonical single nucleotide polymorphism nomenclature. Furthermore, we tested 20 isolates from all main Swiss clades for antimicrobial susceptibility against a panel of antimicrobial agents. All 52 sequenced isolates from Switzerland belong to major clade B.6, specifically subclades B.45 and B.46, previously described in Western Europe. We were able to accurately reconstruct the population structure according to the global phylogenetic framework. No resistance to clinically recommended antibiotics could be identified *in vitro* or *in silico* in the western B.6 strains.

## 1. Introduction

Tularemia is a disease affecting animals and humans and is caused by *Francisella tularensis*. This is a facultative intracellular bacterium that infects and proliferates in macrophages and other phagocytic cells of various hosts. Due to its high virulence, low infectious dose, easy aerosol dispersal, and historical development as a biological weapon, it is classified as a risk group 3 pathogen and a biological threat (Dennis et al., 2001). Humans contract tularemia from rabbits, rodents, biting insects, ticks, and the inhalation of infectious aerosols. Infection can cause various forms of disease in adults and children depending on the subspecies of the bacterium and the route of infection (Tärnvik and Berglund, 2003).

The two clinically relevant subspecies, *F. tularensis* subsp. *tularensis (Ftt)* and *holarctica (Fth)*, differ in their geographical distribution, virulence, and severity of disease. The more virulent *Ftt* is found only in North America (Guryčová, 1998; Vogler et al., 2009a) and is associated with fatal pulmonary infections, whereas the less virulent *Fth* is distributed throughout the Northern Hemisphere and was recently found in the Southern Hemisphere (Eden et al., 2017) The other known subspecies, *F. tularensis* subsp. *mediasiatica,* present in Central Asian region and southern Siberia, has low or unknown pathogenicity for humans (Timofeev et al., 2017).

Due to the clonal evolution of *Fth*, canonical single nucleotide polymorphisms (canSNPs) can be used to robustly designate sublinages. Therefore, *Fth* is currently subdivided into four major clades B.4, B.6, B.12, and B.16 (Svensson et al., 2009; Vogler et al., 2009b). In Europe, most *Fth* strains belong to the basal clades B.6 and B.12. The erythromycin-susceptible clade B.6 is found in Western Europe and North America and corresponds to biovar I, whereas clade B.12 was isolated mainly in Eastern Europe and correlates with erythromycin-resistant biovar II (J et al., 2009; Svensson et al., 2009; Karlsson et al., 2013). Both major clades are found in Switzerland, Germany, Sweden, Norway, and Finland (Svensson et al., 2009; Karlsson et al., 2013; Müller et al., 2013; Wittwer et al., 2018). A study on bacterial isolates from wildlife, humans, and ticks found an high genetic diversity of *Fth* in Switzerland compared to its neighboring countries (Dwibedi et al., 2016; Wittwer et al., 2018). Therefore, Switzerland may represent an expansion origin for founder populations of the western B.6 clade, which then spread to the rest of Western Europe with a gradual decrease in genetic diversity (Dwibedi et al., 2016). A better understanding of the phylogenetic relationships associated with the pathogenicity of specific clades is needed to correlate genetics with environmental and animal reservoirs, vectors, and transmission routes.

Clinical phenotypes of tularemia have a broad differential diagnosis often leading the clinicians to use ineffective antibacterials such as β-lactams, to which *Francisella tularensis* is intrinsically resistant (Buettcher and Imbimbo, 2021). The pathogen’s facultative intracellular life cycle poses additional challenges to systemic antibiotic therapy that can lead to treatment failures. Therapeutic options for tularemia are henceforth limited and no vaccine is currently licensed for human use. Concern has arisen that resistance may develop to currently available treatments. Current treatment guidelines recommend aminoglycosides (e.g., streptomycin or gentamicin), fluoroquinolones (e.g., ciprofloxacin), and tetracyclines (e.g., doxycycline; Dennis et al., 2001; Caspar and Maurin, 2017). However, cure rates vary depending on the antibiotic used, the time to initiation and duration of appropriate antibiotic therapy, and the occurrence of complications. Determining whether a strain is antimicrobial resistant is crucial for determining effective treatment. While most techniques rely on cultures, recent studies have identified multiple protein expression patterns in both antimicrobial resistant (AMR) and susceptible (AMS) strains of *F. tularensis* and *Yersinia pestis* which could indicate conserved phenotypic features of AMR (Deatherage Kaiser et al., 2022).

Although the prevalence of reported infections with *Francisella tularensis* is low, the incidence has increased in the last few years, and outbreaks have become more frequent in recent years in Europe and the United States (Eliasson et al., 2002; Reintjes et al., 2002; Brett et al., 2014; Dupont et al., 2015).

The aim of this study is to characterize the current biodiversity of *Fth* in Switzerland. Therefore, isolates collected from humans from the last 5 years and from *Ixodus ricinus* ticks between 2009 and 2019 were whole-genome sequenced, genotyped, and compared to all previously published European and Swiss *Fth* isolates. These updated phylogenetic relationships between isolates, together with surveillance data of reported human tularemia cases and *in vitro* antibiotic sustainability testing of 20 Swiss *Fth* isolates against a large panel of antibiotics, provide new insights into the epidemiology of tularemia and verify the absence of acquired resistance to first-line drugs in Switzerland.

## 2. Methods

### 2.1. Isolates

This study examined a collection of strains isolated mainly from clinical human biopsies. The samples were first tested for tularemia in various laboratories before the isolates were forwarded to the National Reference Laboratory for Tularemia at Spiez Laboratory, Spiez, Switzerland, for confirmation of diagnosis and storage in the culture collection. The sequences of 49 human and 4 tick isolates collected during 2009–2022 had not been previously characterized and were compared to previously published isolates from humans, animals, and ticks in Switzerland and Europe (Supplementary Table 1; Dwibedi et al., 2016; Wittwer et al., 2018; Kittl et al., 2020).

### 2.2. Whole-genome sequencing and bioinformatic analysis

The *Fth* strains used in the present study were cultured on Polyvitex^®^ agar plates (bioMérieux, Marcy-l’Étoile, France) at 37°C and 5% CO_2_ under biosafety level 3 laboratory conditions. Bacterial cells were harvested after 48 h incubation, and DNA was extracted and purified with the QIAGEN DNeasy blood and tissue kit (Qiagen, Hilden, Germany). The quantity was examined with a Qubit 2.0 fluorometer (Life Technologies, Darmstadt, Germany). A library was prepared with the TruSeq DNA PCR-free kit and sequenced at the NGS platform of the University of Bern, Bern, Switzerland, on a NovaSeq6000 instrument (Illumina Inc., San Diego, CA, United States), which produced 2 × 150 bp paired-end read data.

Data quality was assessed using FastQC v0.11.9 (Babraham Bioinformatics – FastQC A Quality Control Tool for High Throughput Sequence Data, n.d.), and quality trimming was performed with Fastp v0.23.2 (Chen et al., 2018). Kraken2 v2.1.2 software (Wood et al., 2019) was used to check for possible contamination. Genomes were assembled *de novo* using SPAdes v3.15.4 (Bankevich et al., 2012) and contigs shorter than 200 bp were removed using SeqKit v2.2.0 (Shen et al., 2016). Genomic metrics were checked using QUAST v5.0.2 (Gurevich et al., 2013) and BUSCO v5.3.2 (Manni et al., 2021). The whole-genome Average Nucleotide Identity (ANI) was computed using FastANI v1.33 (Jain et al., 2018) between the assemblies and the French-Iberian *Fth* FTNF002-00 genome (NC_009749) as reference. Core genome was inferred with Roary v3.12.0 (Page et al., 2015) using a 95% blastp identity threshold with a 100% identity. Using this generated core genome alignment, the maximum likelihood phylogeny were inferred using IQ-TREE v2.2.03 (model of nucleotide substitution: K3Pu + F + I; Bootstrap replicates:1000; Kalyaanamoorthy et al., 2017; Minh et al., 2020). The R package ggtree v3.4.4 (Yu et al., 2017) was used to build the phylogenetic tree and the tree was rooted against the B.12 outgroup.

Abricate v1.0.1 (tseemann/abricate: Mass Screening of Contigs for Antimicrobial and Virulence Genes, n.d.) was used to infer genes associated with antibiotic resistance with the databases ARG-ANNOT (Gupta et al., 2014), CARD (Jia et al., 2017), EcOH (Ingle et al., 2016), NCBI Bacterial Antimicrobial Resistance Reference Gene Database (Accession: PRJNA313047), and ResFinder (Zankari et al., 2012) with a minimum identity of 50%. CanSNPer v2.0.6 (Lärkeryd et al., 2014) was used to assign the established canSNP nomenclature to the sequenced strains.

### 2.3. Antimicrobial susceptibility testing

To assess the antibiotic susceptibility of 20 *Fth* isolates, the minimum inhibitory concentration (MIC) of the *Fth* to a range of freeze-dried antimicrobial agents was measured on MICRONAUT test plates (Merlin, Bornheim-Hersel, Germany). The antimicrobials tested on the MICRONAUT plate were amoxicillin/clavulanic acid, vancomycin, rifampicin, ciprofloxacin, levofloxacin, doxycycline, tetracycline, gentamicin, streptomycin, chloramphenicol, trimethroprim/sulfamethoxazole, linezolid, azithromycin, ceftazidime, imipenem, meropenem, ceftriaxone, erythromycin, and moxifloxacin and were determined according to Clinical and Laboratory Standards Institute (CLSI) and WHO guidelines.

The AST of *Fth* strains was performed with cation-adjusted Mueller-Hinton broth enriched with 2% defined growth additive Polyvitex^®^ (bioMérieux, Marcy-l’Étoile, France). The bacterial inoculum was calibrated to a final concentration of 5 × 10^5^ CFU/mL. The culture media were incubated for 48 h in an atmosphere enriched with 5% CO_2_, and the MICs were interpreted using the CLSI or EUCAST susceptibility breakpoints for *Francisella tularensis* (Methods for Antimicrobial Dilution and Disk Susceptibility Testing of Infrequently Isolated or Fastidious Bacteria, n.d.). The *Escherichia coli* strain ATCC 25922 was used as a control.

### 2.4. Surveillance data

All human and animal surveillance data described was kindly provided by the Federal Office for Public Health (FOPH), Liebefeld, Switzerland. Note that tularemia cases can have a reporting delay of several months likely leading to a small underreporting of cases in the autumn and winter of 2022. Moreover, the FOPH only supplied patient residency data, hence only this information was utilized for geographic representation of the surveillance data.

## 3. Results

### 3.1. Whole-genome sequencing data

Genome sequencing of the 52 isolates analyzed in this study yielded an average of 8,245,642 reads per isolate with an average coverage of 1,304-fold. Genome assembly revealed an average genome size of 1,778,088 bp with a minimum of 1,777,484 bp and maximum of 1,781,251 bp. The GC-content is 32% in all the isolates. The mean N50 value was 26.04 kbp. The assemblies are available in BioProject PRJNA841431. No contamination was found with Kraken2 in any of the strains (Table 1).

**Table 1:** Overview of the 52 whole-genome sequenced *Fth* strains from Switzerland used in this study.

Isolate	Accession number	Country	Source	Year	Subclade	Terminal clade	Number of reads	GC content (%)	Dublicated reads (%)	Whole genome coverage
Genomes made public through this work (*n* = 52)										
FT101	SAMN28593779	Switzerland	Human	2019	B.45	B.92	8,393,174	32	58.24	1328.04
FT102	SAMN28593780	Switzerland	Human	2019	B.45	B.91	9,219,940	32	61.86	1458.86
FT103	SAMN28593781	Switzerland	Human	2019	B.45	B.88	9,294,314	32	62	1470.62
FT104	SAMN28593782	Switzerland	Human	2019	B.45	B.49	10,022,969	32	63.18	1585.92
FT105	SAMN28593783	Switzerland	Human	2019	B.45	B.262	6,432,314	32	54.33	1017.77
FT106	SAMN28593784	Switzerland	Human	2019	B.45	B.53	7,068,151	32	55.97	1118.38
FT107	SAMN28593785	Switzerland	Tick	2019	B.45	B.45	7,439,341	32	57.19	1177.11
FT108	SAMN28593786	Switzerland	Tick	2019	B.45	B.45	7,895,506	32	60.4	1249.29
FT109	SAMN28593787	Switzerland	Human	2019	B.45	B.284	7,312,196	32	58.05	1,157
FT111	SAMN28593789	Switzerland	Human	2019	B.45	B.51	6,932,406	32	55.46	1096.9
FT112	SAMN28593790	Switzerland	Human	2019	B.45	B.53	8,698,531	32	58.97	1376.35
FT113	SAMN28593791	Switzerland	Human	2020	B.45	B.62	6,174,406	32	50.38	976.97
FT114	SAMN28593792	Switzerland	Human	2020	B.45	B.53	6,945,375	32	54.48	1098.96
FT115	SAMN28593793	Switzerland	Human	2020	B.46	B.63	8,616,036	32	59.86	1363.3
FT116	SAMN28593794	Switzerland	Human	2020	B.45	B.49	6,955,593	32	53.28	1100.57
FT117	SAMN28593795	Switzerland	Human	2020	B.46	B.47	7,512,674	32	53.75	1188.72
FT118	SAMN28593796	Switzerland	Human	2020	B.45	B.92	9,157,625	32	60.77	1,449
FT119	SAMN28593797	Switzerland	Human	2021	B.45	B.92	10,623,849	32	63.22	1680.99
FT120	SAMN28593798	Switzerland	Human	2021	B.45	B.284	7,735,822	32	57.39	1224.03
FT121	SAMN28593799	Switzerland	Human	2021	B.45	B.53	8,499,093	32	62.15	1344.8
FT123	SAMN28593800	Switzerland	Human	2021	B.45	B.92	6,540,565	32	56.69	1034.9
FT124	SAMN28593801	Switzerland	Human	2021	B.46	B.47	6,758,707	32	56.36	1069.42
FT125	SAMN28593802	Switzerland	Human	2021	B.46	B.46	6,444,786	32	54.3	1019.75
FT126	SAMN28593803	Switzerland	Human	2021	B.45	B.51	7,207,443	32	57.69	1140.42
FT127	SAMN28593804	Switzerland	Human	2022	B.45	B.45	6,339,254	32	55.86	1003.05
FT128	SAMN28593805	Switzerland	Human	2022	B.45	B.49	12,894,091	32	69.72	2040.21
FT129	SAMN28593806	Switzerland	Human	2022	B.45	B.45	5,194,050	32	53.26	821.85
FT130	SAMN28593807	Switzerland	Human	2022	B.45	B.55	9,081,012	32	63.84	1436.87
FT131	SAMN28593808	Switzerland	Human	2022	B.45	B.62	7,589,266	32	60.69	1200.84
FT132	SAMN28593809	Switzerland	Human	2022	B.45	B.45	15,928,471	32	75	2520.34
FT133	SAMN28593810	Switzerland	Human	2022	B.45	B.53	10,661,254	32	66.71	1686.91
FT134	SAMN28593811	Switzerland	Human	2022	B.86	B.86	9,741,684	32	65.9	1541.41
FT136	SAMN28593813	Switzerland	Human	2022	B.45	B.90	10,320,185	32	66.46	1632.95
FT137	SAMN28593814	Switzerland	Human	2022	B.46	B.47	7,694,824	32	59.73	1217.54
FT76	SAMN28593756	Switzerland	Human	2015	B.45	B.55	9,734,227	32	62.92	1540.23
FT77	SAMN28593757	Switzerland	Human	2015	B.45	B.284	7,665,291	32	59.3	1212.87
FT78	SAMN28593758	Switzerland	Tick	2009	B.45	B.62	7,842,078	32	57.14	1240.84
FT79	SAMN28593759	Switzerland	Tick	2009	B.45	B.45	8,890,993	32	60.23	1406.81
FT82	SAMN28593761	Switzerland	Human	2016	B.45	B.251	6,575,408	32	53.47	1040.42
FT83	SAMN28593762	Switzerland	Human	2016	B.45	B.284	6,327,548	32	53.69	1001.2
FT84	SAMN28593763	Switzerland	Human	2016	B.45	B.88	8,088,271	32	57.33	1279.79
FT85	SAMN28593764	Switzerland	Human	2016	B.46	B.46	9,576,863	32	63.8	1515.33
FT87	SAMN28593765	Switzerland	Human	2017	B.45	B.246	7,916,254	32	59.11	1252.58
FT88	SAMN28593767	Switzerland	Human	2017	B.45	B.284	7,054,550	32	57.07	1116.23
FT91	SAMN28593769	Switzerland	Human	2017	B.45	B.45	5,686,891	32	53.36	899.83
FT92	SAMN28593770	Switzerland	Human	2017	B.45	B.92	7,547,124	32	55.58	1194.17
FT93	SAMN28593771	Switzerland	Human	2018	B.45	B.91	7,248,895	32	56.18	1146.98
FT94	SAMN28593772	Switzerland	Human	2018	B.46	B.47	10,817,838	32	66.5	1711.69
FT95	SAMN28593773	Switzerland	Human	2018	B.45	B.53	7,945,810	32	57.76	1257.25
FT96	SAMN28593774	Switzerland	Human	2018	B.45	B.287	10,403,263	32	62.87	1646.09
FT97	SAMN28593775	Switzerland	Human	2018	B.45	B.239	6,249,813	32	53.67	988.9
FT98	SAMN28593776	Switzerland	Human	2018	B.45	B.53	8,750,157	32	60.46	1384.52

In addition, 74 assemblies of strains from Swiss wildlife, humans, and ticks previously described (Dwibedi et al., 2016; Wittwer et al., 2018; Kittl et al., 2020) were downloaded from NCBI and included for comparison. Moreover, to put the newly sequenced strains into the context of the European phylogenetic framework 15 assemblies with a source information of Europe was downloaded from NCBI and included into the phylogenetic tree (Supplementary Table 1).

### 3.2. *Francisella tularensis* subspecies *holarctica* phylogeography in Switzerland

**Figure 1:**
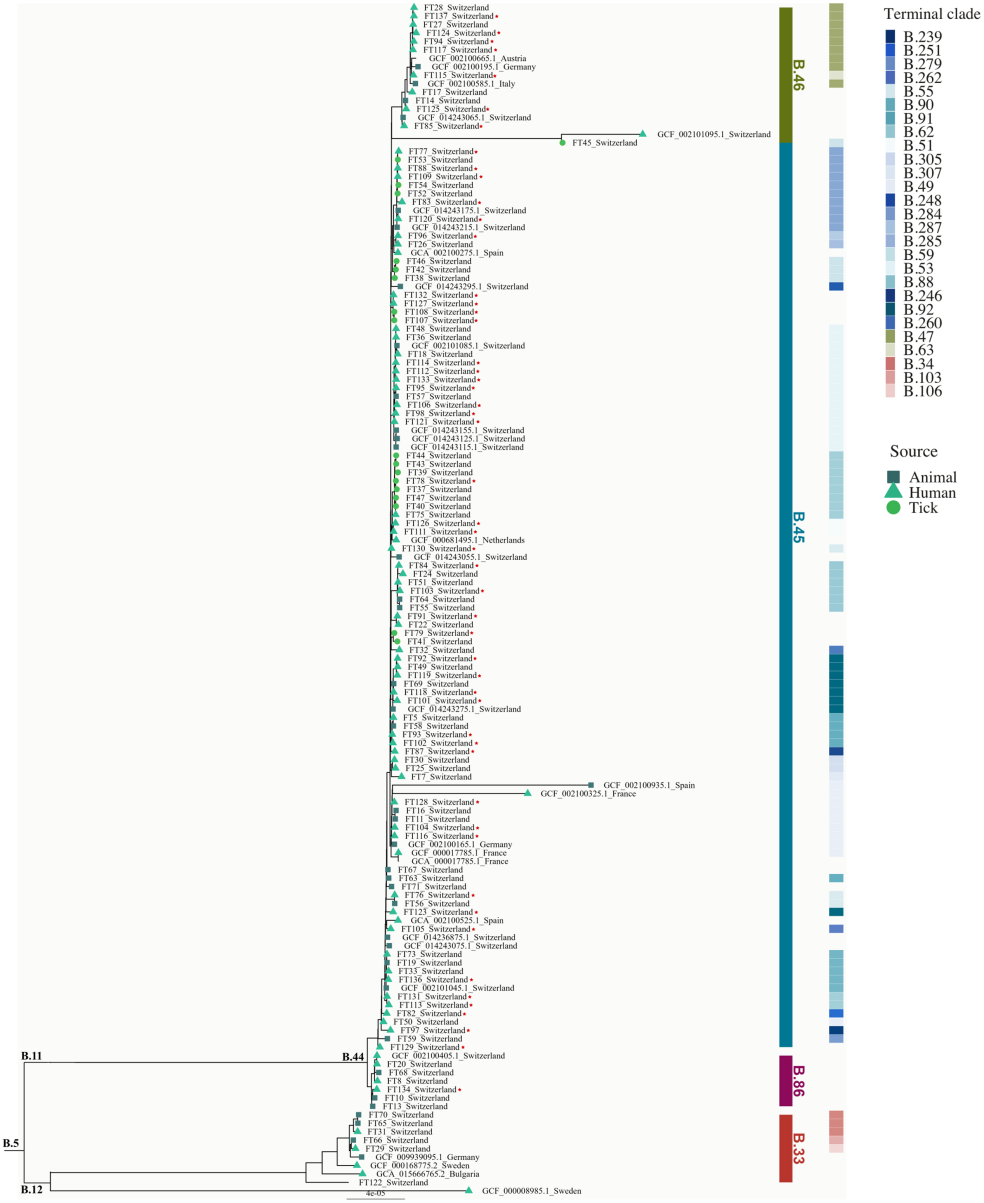
Phylogeny of Swiss *Fth* isolates. Phylogeny of *Fth* isolates (*n* = 141) obtained from tick, animal, and human hosts (tick: circle; animal: rectangle; human: triangle). Reference genomes (*n* = 15) from different European countries were included. Clades (B.33, B.86, B.45, and B.46) and terminal clades were labeled according to the canSNP nomenclature. The newly sequenced isolates (*n* = 52) are marked with a star. The tree was rooted with the B.12 as out-group. The scale bar corresponds to the average number of substitutions per site.

A phylogenetic tree of all 126 Swiss (52 newly sequenced +74 downloaded accessions) and 15 European isolates is shown in Figure 1. The isolates have an average nucleotide identity of >99.9% compared to the *Fth* reference strain FTNF002-00.

**Figure 2:**
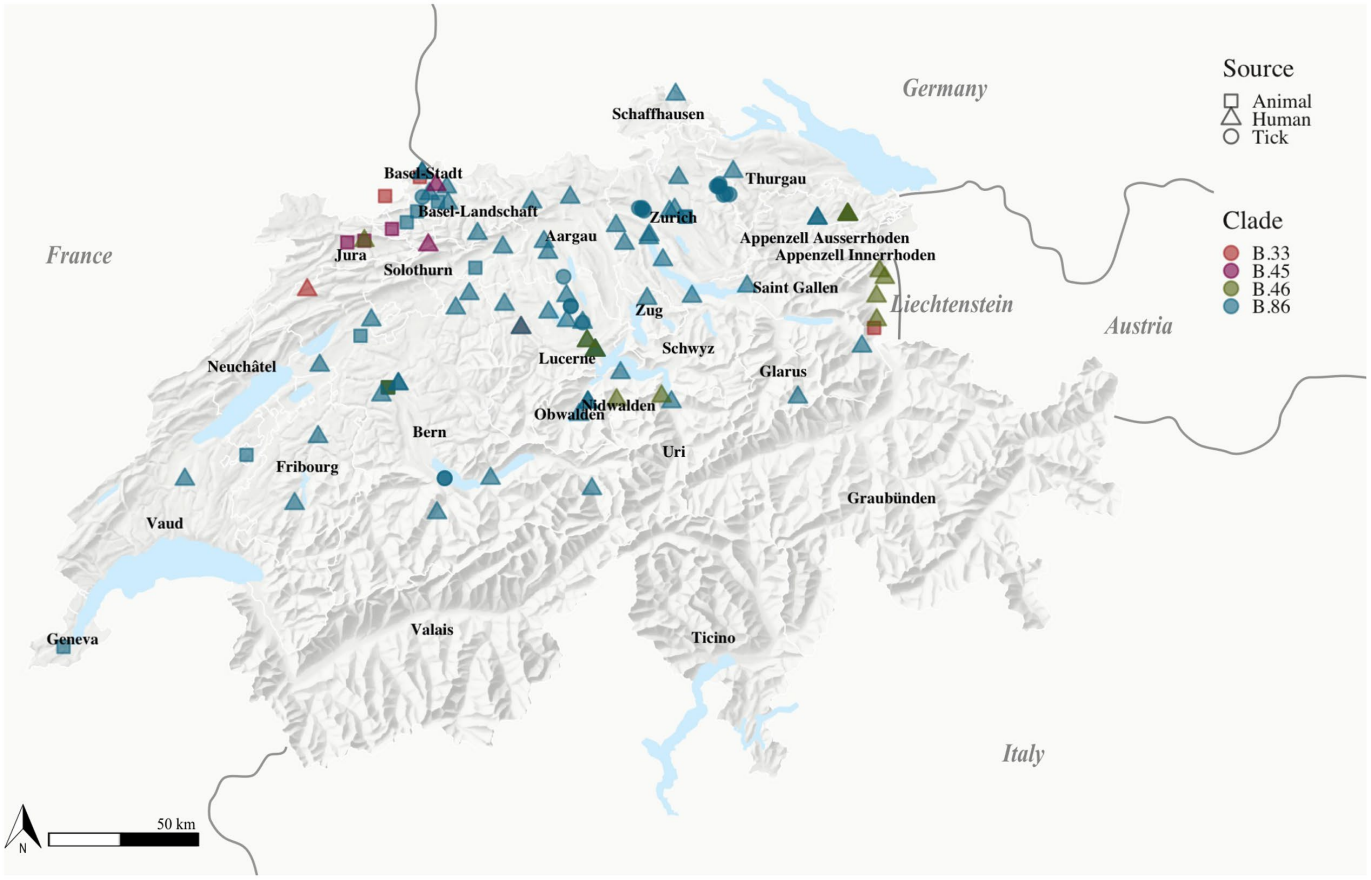
Location of *Fth* isolates. *Fth* isolates (*n* = 111) with known geographic locations obtained from tick (*n* = 18), animal (*n* = 20), and human (*n* = 73) sources labeled according to their clade [B.33 (*n* = 5), B.86 (*n* = 5), B46 (*n* = 12), B45 (*n* = 89)], Switzerland, 2007–2022. The geographical location of the isolates obtained from human sources was represented by the patients’ residence.

Using CanSNPer2, the SNP status of the isolates was retrieved using all currently available canonical SNP markers of *Fth* (*n* = 301). Of the 126 Swiss isolates analyzed, the majority (*n* = 121) belonging to the major clade B.6 grouped together with the reference genomes from France, Spain, Italy, Liechtenstein, Austria, Germany, Netherlands and Sweden. 5 Swiss isolates clustered together with genomes from Sweden, Germany and Bulgaria. None of the newly sequenced 52 isolate belong to the Northeast European B.12 clade (Figure 1). Seven isolates belong to the newly described B.6 subclade B.86 that can so far only be found in Switzerland (Wittwer et al., 2018). One of the newly sequenced human isolates belongs to this clade and was found in the same geographic region of Basel-Land as the isolates already described (Figure 2).

The B.6 subclade B.44 can be further subdivided into the subclades B.45 and B.46. Subclade B.45 was represented by the majority of samples (*n* = 101): 45 of the newly sequenced samples belong to this clade and were located all over the northern part of Switzerland. All four recently genotyped strains obtained from ticks belong to subclade B.45.

Subclade B.46 was represented by 14 isolates with 7 newly sequenced human isolates and was found as previously described in the two regions of the Jura and Lucerne (Wittwer et al., 2018).

### 3.3. Antimicrobial susceptibility testing of Swiss *Fth* isolates

The MIC values of 20 *Fth* isolates obtained from 13 human, 4 animal, and 3 tick hosts for a variety of antimicrobial agents are shown in Supplementary Table 2.

All strains tested were sensitive to the antibiotic classes recommended for first-line treatment of tularemia. The MICs for the aminoglycosides tested ranged from 0.25 to 0.5 mg/L for gentamicin and from 2 to 4 mg/L for streptomycin. The fluoroquinolones ciprofloxacin (MIC range: 0.0625–0.125 mg/L), levofloxacin (MIC range: 0.0625–0.125 mg/L) and moxifloxacin (MIC: 0.25 mg/L) were the most active compounds *in vitro*. All strains tested had MIC values for these antibiotics that were at least four times below the CLSI threshold for susceptibility. The MICs for doxycycline and tetracycline were 0.25 mg/L.

As expected, none of the β-lactams tested, amoxicillin/clavulanic acid, ceftazidime, ceftriaxone, imipenem, or meropenem, showed any bactericidal activity against *Fth*. Although β-lactams are classically considered bactericidal drugs against most other bacteria, they are considered unreliable for the treatment of tularemia (Tärnvik and Chu, 2007).

For the macrolides, 18 isolates of B.6 subclade (biovar I) were susceptible to erythromycin (MIC range: 2–4 mg/L), and two isolates FT29 and FT65 clustered to the B.12 genotype (biovar II) were resistant (MIC: > 32 mg/L). In addition, the assemblies were screened for antimicrobial resistance and virulence genes. It was discovered that all isolates belonging to the B.12 subclade (biovar II) contained a single mutation, A2059C, in the three copies of the *rrl* gene, encoding the 23S rRNA. This mutation has been associated with erythromycin resistance in biovar II, as previously described by Karlsson et al. (2016). No further associations were found through *in silico* analysis. The MIC ranges of azithromycin were similar to those of erythromycin and showed the same dichotomy between genotype B.6 (biovar I) and genotype B.12 (biovar II).

### 3.4. Tularemia in Switzerland

Tularemia has been a notifiable disease for humans and animals in Switzerland since 2004. To identify disease and determine the source of infection, clinical symptoms compatible with tularemia and one laboratory criterion must be reported to the FOPH by the attending physician. A positive laboratory finding is the isolation of the live pathogen by cultivation or the serological detection of specific antibodies or specific nucleic acids. Tularemia is most likely underdiagnosed and underreported because the disease is generally rare and patients presenting with it are rarely diagnosed because of non-disease-specific clinical symptoms.

**Figure 3:**
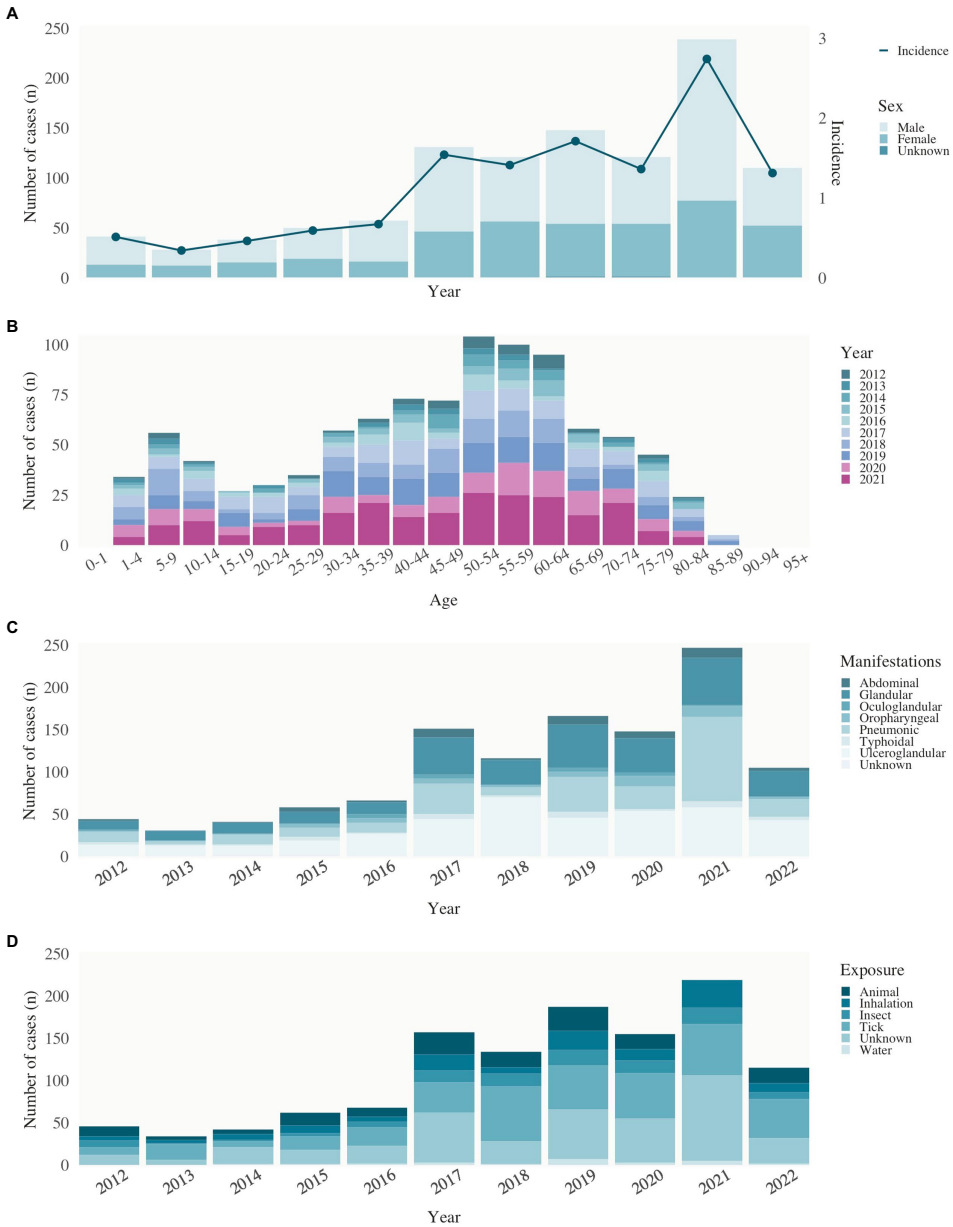
Tularemia epidemiology in human. **(A)** Human notified cases of tularemia (stacked bars) of male, female, and unknown sex and incidence (per 100,000 population, second *y*-axis) by year of notification, Switzerland, 2012–2022. **(B)** Age distribution of the reported cases by year of notification, Switzerland, 2012–2022. **(C)** Clinical manifestations of reported cases by year of notification, Switzerland, 2012–2022. **(D)** Source of infection of reported cases by year of notification, Switzerland, 2012–2022.

Between 2012 and 2022, a total of 1,081 cases were registered, corresponding to a mean annual incidence of 1.14 cases per 100,000 inhabitants and a male-to-female ratio of 1.7 to 1 (Figure 3A). While an average of 43 cases per year were reported from 2012 to 2016, an increase to 130 cases per year was observed from 2017 to 2020. In 2021, a further increase of cases was reported to 239, corresponding to an annual incidence of 2.71 (2020: 121; 2021: 239) cases per 100,000 population, with a male-to-female ratio of 2.1 to 1, representing a 2.4-fold increase in the number of reported male cases. By December 2022, 100 tularemia cases had been reported, that indicates a decrease back to the levels of 2020.

Tularemia is known to occur in patients aged 1–89 years. An initial peak of cases is observed in the age group of 5–9 years. Reporting rates increase with age, peaking between 50 and 64 years of age and decreasing above 65 years of age (Figure 3B).

**Figure 4:**
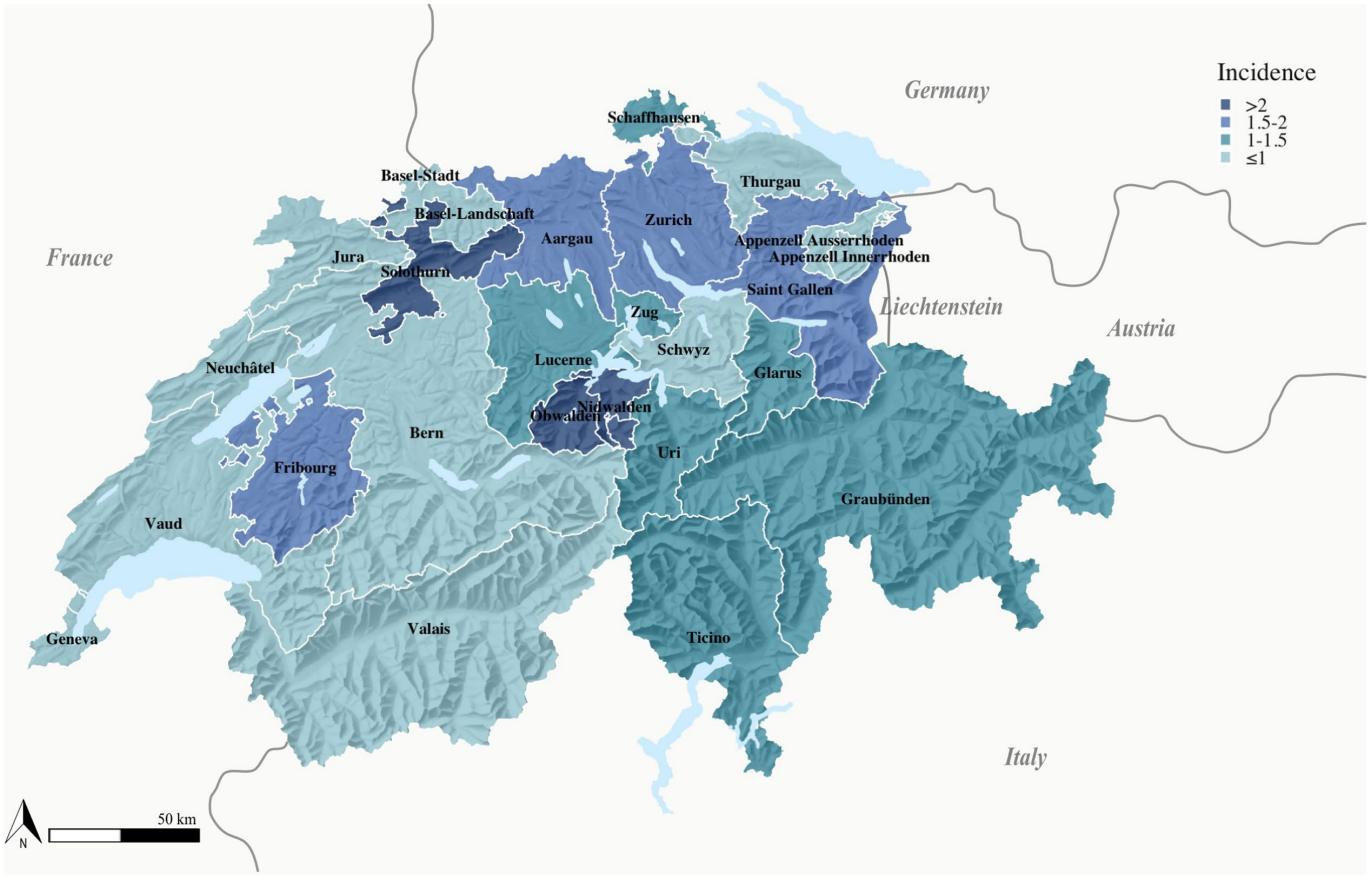
Map of tularemia incidence in human. Mean tularemia incidence per 100,000 inhabitants, Switzerland, 2012–2022. Only the residence of the patients was available for display of geographical distribution.

In the cases reported in Switzerland between 2012 and 2022, the most common clinical manifestation (60% of cases) was glandular and ulceroglandular tularemia, typically associated with the bite of a hematophagous arthropod. The pulmonary type was present in 25% and abdominal/oropharyngeal type in 9% of patients. From 2020 to 2021, the number of pulmonary manifestations doubled from 18 to 40%, before decreasing back to 20% in 2022 (Figure 3C). These clinical manifestations are consistent with the reported sources of infection. The majority of patients (40%) reported an insect or arthropod bite prior to the onset of tularemia symptoms. In 15%, the cause of infection was suspected to be contact with wild animals and for 32% of patients the source of infection was not known (Figure 3D). Between 2012 and 2022, the highest mean annual incidences were recorded in local clusters in the cantons of Obwalden, Nidwalden and Solothurn (Figure 4).

**Figure 5:**
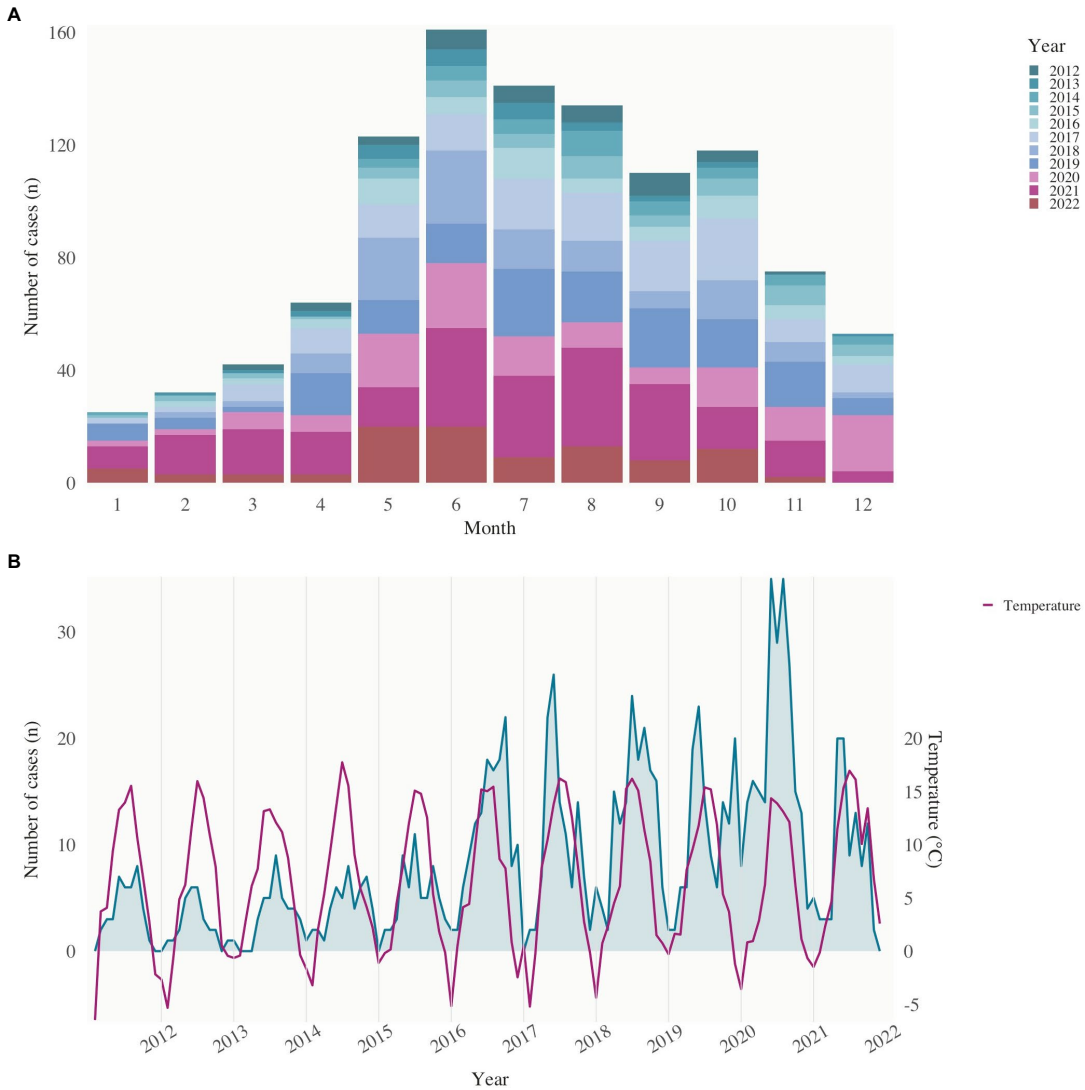
Seasonality of reported human tularemia cases. **(A)** Notified cases of tularemia (stacked bars) by month of notification, Switzerland, 2012–2022. **(B)** Notified cases of tularemia from 2012 to 2022 and mean temperature (°C, second *y*-axis) in Switzerland.

Tularemia is a seasonal disease in Switzerland, with most patients showing symptoms from May to November (Figure 5A), when reservoir animal populations peak and frequent outdoor activities such as hunting, farming, and hiking increase contact between wildlife and humans. This is consistent with the seasonal occurrence of tularemia cases in Europe. Since 2017, the increase in reported cases has shifted temporally to April. Between 2020 and 2022, tularemia cases were reported throughout the year, and no break in the winter months was observed. This may have been favored by the warm temperatures in the winter of 2019–2020 (Figure 5B).

## 4. Discussion

### 4.1. Tularemia in Switzerland

Switzerland, where tularemia occurs endemically, reports a higher incidence than any of its neighboring countries, except Sweden, which has a similar incidence (2.8 cases per 100,000 inhabitants; The European Union One Health 2021 Zoonoses Report, n.d.). We assume that the increase in recent years is due to a combination of the actual increase in infection pressure and increased sensitivity of the surveillance system, and this assumption is confirmed by the relatively stable proportion of hospital cases.

Since the introduction of mandatory reporting in 2004, the number of tularemia cases in humans has risen from a minimum 4 in 2004 to a maximum of 239 cases in 2021 (Figure 3A). Possible reasons for the rising incidence of the disease are increased infection pressure due to higher occurrence of the pathogen in environmental reservoirs, hosts, or vectors, and more frequent contact between humans and the hosts or vectors. Notably, in 2021, pneumonic tularemia accounted for 25% of cases (Figure 3C), which can result from inhalation of contaminated aerosols or when other forms of tularemia are untreated, and the bacteria can spread through the bloodstream. This rise of pneumonic tularemia can also be observed in neighboring countries like France (21% of cases in 2018 vs. 10% in previous years; Brunet et al., 2021). It is possible that the increased numbers can be attributed to more people spending leisure time and vacations in Swiss natural environments, particularly during the COVID-19 pandemic. Additionally, delayed treatment due to the pandemic may have contributed to the higher incidence of secondary pulmonary tularemia. Furthermore, a changed cyclical pattern in the onset and duration of reported cases has also been observed in the last 10 years. This can be attributed to changing climatic conditions, which not only leads to a change in the active phase of wildlife and an increased occurrence of arthropod vectors but also allows people to remain in natural environments for prolonged periods.

More males than females presented with tularemia infection, with the age group 45–69 years the most affected for both sexes, which is consistent with the demographic age distribution in Switzerland (Figure 3B). This could also reflect the gender distribution of people working outdoors in rural areas.

Although the regional incidence of tularemia varies widely, with regional clusters in the cantons of Obwalden, Nidwalden Lucerne and Solothurn, long-term surveillance data suggest that the pathogen occurs throughout Switzerland (Figure 4). Variations in the number of reported cases from different cantons may be explained by variations in actual disease incidence due to differing exposure risks and infection pressure factors in reporting activity.

### 4.2. Phylogeography of *Francisella tularensis* subsp. *holarctica* genotypes in Switzerland

The genetic diversity of *Fth* in Switzerland has been studied previously, but as the number of human cases has multiplied since the previous publication by Wittwer et al. (2018), an update on the situation in Switzerland was of interest.

All newly sequenced human *Fth* isolated between 2015 and 2022 belonged to the main clade B.6, and none could be assigned to the B.12 clade previously identified at the Swiss border. Strains belonging to the main clade B.6 were isolated throughout northern Switzerland. Although we know from surveillance data that human cases also occur in southern and eastern Switzerland, no sequencing data are available on these isolates. The four newly sequenced isolates collected from ticks all belong to the B.6 subclade B.45, which fits hypothesis of Wittwer et al. (2018) about a possible better adaptation through higher vector competence. The narrow geographical distribution of the relatively new B.6 subclade B.86 previously described was confirmed. Because tularemia has complex epidemiological cycles involving a variety of host species, reservoirs, and vectors, it is plausible that certain biotopes and climatic conditions might influence components of these cycles and thus favor or hinder the spread and infectivity of some clades. These functional similarities require further investigation.

The phylogeographic pattern of tularemia has been proven to be complex, and genotyping with canSNPs also has some limitations in resolution. A high phylogenetic resolution with local surveillance data may help to better understand the infection pathways of human cases and link outbreaks.

### 4.3. No antimicrobial resistance to clinically recommended antibiotics *in vitro* or *in silico* to *Francisella tularensis* subsp. *holarctica* genotypes in Switzerland

No resistance to any of the clinically recommended antibiotics was detected either *in vitro* or *in silico*, which is consistent with previous data. AST data indicate that the fluoroquinolones have the lowest MICs, are potent and rapid bactericides *in vitro*, and are highly effective for curing *Francisella tularensis* infections in infected humans. Since no macrolide-resistant strains of biovar II (B.12 clade) have been identified in the last 5 years, macrolides, particularly azithromycin, may be an alternative for patients infected with erythromycin-susceptible *Fth* strains (B.6 clade, biovar I) and in cases where these clinically recommended treatments are contraindicated, including pregnancies or allergy. The narrow MIC distribution observed for each of the antibiotics tested is consistent with the high genetic similarity between *Fth* strains isolated in Switzerland.

## 5. Conclusion

In conclusion, we have reconfirmed B.45 as the most important clade of *Fth* occurring in Switzerland, and that it does not show antimicrobial resistance to clinically recommended antibiotics *in vitro* or *in silico*.

Cases of tularemia in humans and animals are notifiable in Switzerland and neighboring European countries. To understand the complexity of the disease, close collaboration between veterinary and human medicine and public health in all parts of Switzerland and internationally is essential. As the bacterium can also be transmitted by insects, knowledge of its prevalence in ticks and mosquitoes is of interest. Combining this with the investigation of the prevalence of the pathogen in animal and environmental samples can achieve a better understanding of the pathogen’s transmission cycle.

## Data availability statement

The datasets presented in this study can be found in online repositories. The names of the repository/repositories and accession number(s) can be found in the article/Supplementary material.

## Author contributions

SS conducted the laboratory experiments, analyzed the data, and drafted the manuscript. NL and MM provided expertise in whole-genome phylogeny. RB and MW coordinated and supervised the work. All authors contributed to the article and approved the submitted version.

## Funding

This work was funded by a research grant of the Federal Department of Defense, Civil Protection and Sport (FOCP).

## Conflict of interest

The authors declare that the research was conducted in the absence of any commercial or financial relationships that could be construed as a potential conflict of interest.

## Publisher’s note

All claims expressed in this article are solely those of the authors and do not necessarily represent those of their affiliated organizations, or those of the publisher, the editors and the reviewers. Any product that may be evaluated in this article, or claim that may be made by its manufacturer, is not guaranteed or endorsed by the publisher.
