# Supplementary material for: Phylogeography of Francisella tularensis subspecies holarctica and epidemiology of tularemia in Switzerland
Source: Front Microbiol. 2023 Apr 11;14:1151049. doi: 10.3389/fmicb.2023.1151049 (PMC10126411; doi:10.3389/fmicb.2023.1151049)
Supplement: Supplementary file 1 [file Table_1.DOCX]

Supplementary Material

Phylogeography of *Francisella tularensis* subspecies *holarctica* and Epidemiology of tularemia in Switzerland

**Sara Doina Schütz^1,2,3^, Nicole Liechti^2^,** [**Ekkehard Altpeter**](https://pubmed.ncbi.nlm.nih.gov/?term=Altpeter%20E%5BAuthor%5D)**^4^, Anton Labutin^4^, Tsering Wütrich^2^, Kristina Maria Schmidt^2^, Michael Buettcher^5,6,7^, Michel Moser^2^, Rémy Bruggmann^1^ and Matthias Wittwer^2^**

*** Correspondence:**Matthias Wittwer
Matthias.wittwer@babs.admin.ch

*Supplementary Table 1: Overview of the additional previously published 74 Swiss and 15 European Fth isolates used in this study.*

*
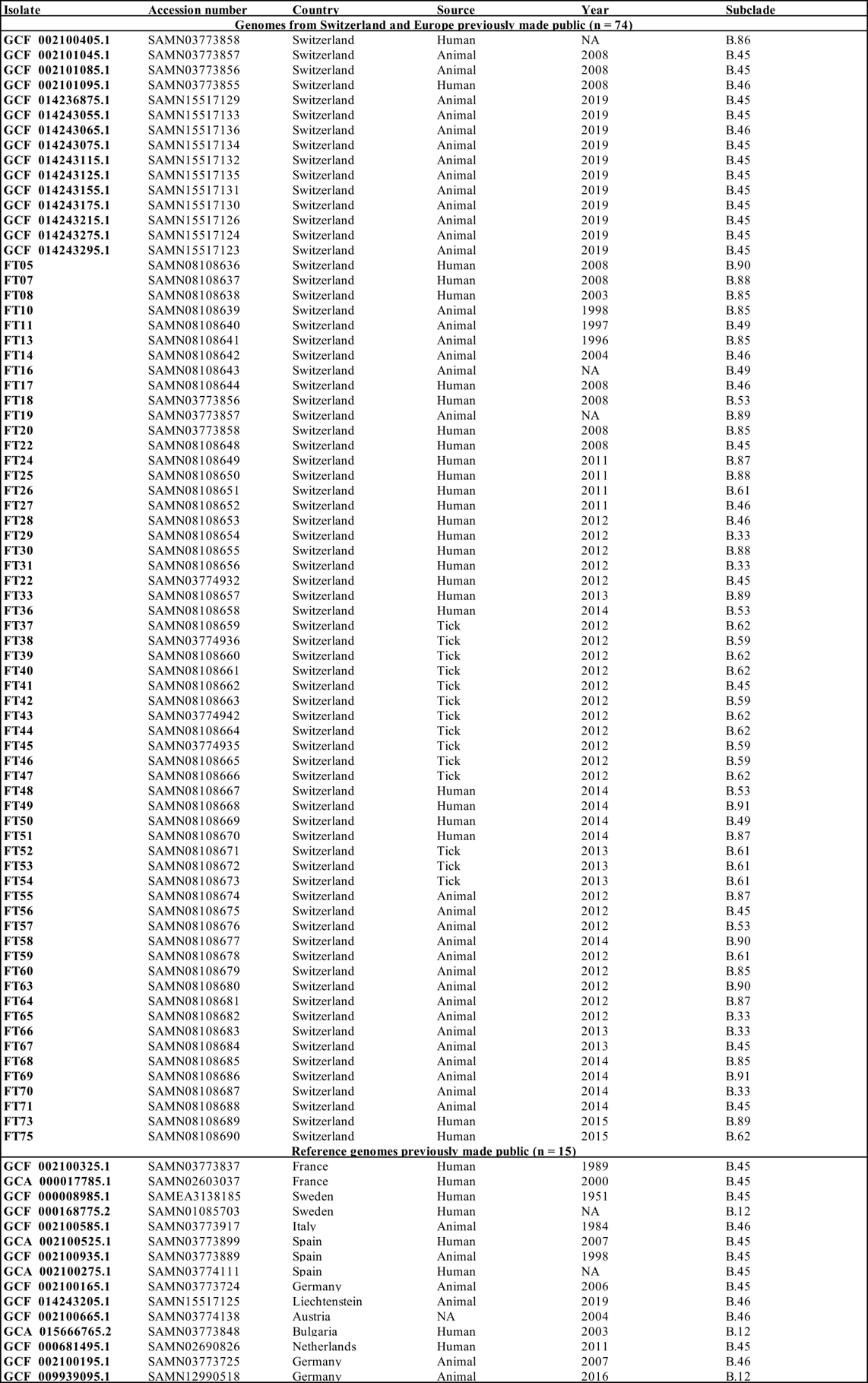
*

*Supplementary Table 2: MIC results (mg/L) for 20 Fth strains. S: susceptible; R: resistant; a: CLSI breakpoints; b: EUCAST PK/PD breakpoints; c: H. influenzae EUCAST breakpoints.*

*
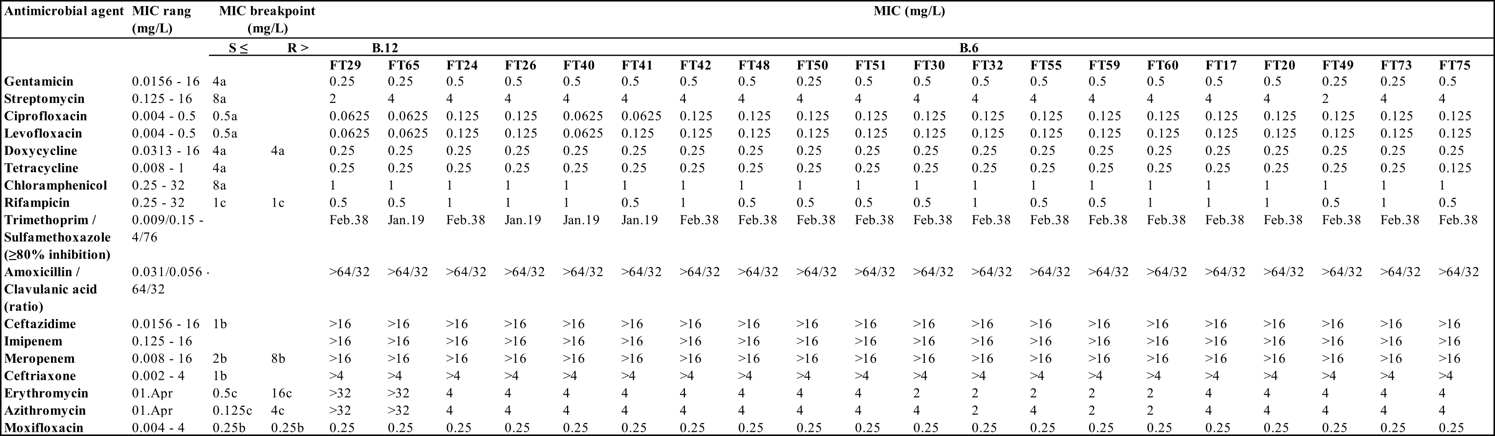
*
